# NMR-Based Metabolomic Profiling of Overweight Adolescents: An Elucidation of the Effects of Inter-/Intraindividual Differences, Gender, and Pubertal Development

**DOI:** 10.1155/2014/537157

**Published:** 2014-03-27

**Authors:** Hong Zheng, Christian C. Yde, Karina Arnberg, Christian Mølgaard, Kim F. Michaelsen, Anni Larnkjær, Hanne C. Bertram

**Affiliations:** ^1^Department of Food Science, Aarhus University, Kirstinebjergvej 10, 5792 Aarslev, Denmark; ^2^Department of Human Nutrition, Faculty of Life Sciences, University of Copenhagen, 1958 Frederiksberg, Denmark

## Abstract

The plasma and urine metabolome of 192 overweight 12–15-year-old adolescents (BMI of 25.4 ± 2.3 kg/m^2^) were examined in order to elucidate gender, pubertal development measured as Tanner stage, physical activity measured as number of steps taken daily, and intra-/interindividual differences affecting the metabolome detected by proton NMR spectroscopy. Higher urinary excretion of citrate, creatinine, hippurate, and phenylacetylglutamine and higher plasma level of phosphatidylcholine and unsaturated lipid were found for girls compared with boys. The results suggest that gender differences in the metabolome are being commenced already in childhood. The relationship between Tanner stage and the metabolome showed that pubertal development stage was positively related to urinary creatinine excretion and negatively related to urinary citrate content. No relations between physical activity and the metabolome could be identified. The present study for the first time provides comprehensive information about associations between the metabolome and gender, pubertal development, and physical activity in overweight adolescents, which is an important subject group to approach in the prevention of obesity and life-style related diseases. While this study is preliminary, these results may have the potential to translate into clinical applicability upon further investigations; if biomarkers for Tanner stage can be established, these might be used for identification of individuals susceptible to an early pubertal development.

## 1. Introduction

Metabolomics is a postgenomic technology that is given great promise for human phenotyping and for assisting in health assessment [1, 2]. Numerous metabolomics studies have investigated the impact of anthropometric factors such as age, gender, and obesity [3–5] in an attempt to understand the human metabolome and interindividual differences. However, these studies have mainly been conducted on either newborns and infants [6, 7] or adults [3–5, 8], whereas studies on children and adolescents are sparse [9–11]. Gu et al. [9] studied the age-related metabolic changes in children of age from newborn to 12 years and by NMR-based metabolomics on urine samples. An age effect on the urinary metabolome was identified as a distinct age-dependent clustering in PCA. Metabolites found to be correlated with age included creatinine, creatine, glycine, betaine/TMAO, citrate, succinate, and acetone. While creatinine increased with age, all the other metabolites decreased [9].

The increase in obesity has been much more pronounced in children and adolescents than other age groups [12], and the prevalence of cardiovascular disease (CVD) among youth is also increasing [13]. In fact, many life-style related diseases are assumed to be commenced already in childhood and during adolescence [14]. Thus, it was recently shown that the level of plasma branched-chain amino acids, which are getting increasing attention because of their potential role in insulin sensitivity and secretion, was elevated already in obese children aged 8 to 13 years and correlated with insulin resistance determined 18 months later [15]. Tanner stage was first defined by Marshall and Tanner [16, 17] as a scale of physical development based on external primary and secondary sex characteristics. Oldehinkel et al. [18] investigated the relationship between specific mental health problems and pubertal stage in adolescents in a Dutch prospective cohort study and revealed that Tanner stage was positively related to tiredness, irritability, rule-breaking behaviors, and substance use and negatively to fears and somatic complaints. Consequently, Tanner stage seems to be important for health and well-being, and it could be advantageous to obtain a better understanding of the metabolome of adolescents and relation to pubertal development and life-style related factors.

However, to our knowledge, no metabolomics studies on pubertal development have been reported. Pubertal development involves complex physical and psychological processes between childhood and adult life, ultimately resulting in the attainment of adult reproductive capacity [19]. The past decades age at sexual maturation has declined, evident by a gradual younger age at menarche [20, 21] and breast development [22] among girls, and earlier testicular development in boys [23]. The obesity epidemic is thought to affect timing of pubertal development, and the process of pubertal development is a critical period for body composition development [24], but the association between obesity and pubertal development is far from clear [19]. Consequently, taken the increasing obesity epidemics into consideration, this urges us to learn more about of the process of pubertal development and metabolism, and metabolomics may be a useful tool. Therefore, by using nuclear magnetic resonance- (NMR-) based metabolomics, the aim of the present study was to investigate the plasma and urine metabolome of overweight adolescents and elucidate intra- and interindividual differences, the influence of gender, pubertal development measured as Tanner stage, and physical activity.

## 2. Materials and Methods

### 2.1. Subjects

The samples used in present study included a subset of samples from a larger intervention study presented in Arnberg et al. [25, 26]. A total of 203 overweight adolescents aged 12–15 years with the BMI (25.4 ± 2.3 kg/m^2^) corresponding to a BMI > 25 kg/m^2^ for adults [27] were recruited in the Copenhagen area using extractions from the Civil Registration System. Firstly, a subgroup of 28 subjects was established and a urine sample and a blood sample were collected from each of these at time point 0. These samples served for studies on intra-/interindividual differences. Twelve weeks later, urine and blood samples were collected from all the 203 subjects, and sample sets from 192 subjects were included in the present metabolomics study. All participants were free to consume their usual diet ad libitum and maintain daily physical activity. Samples were frozen and stored at −80°C until analysis.

### 2.2. NMR Measurements

^1^H NMR spectra were measured at 600.13 MHz for proton on a Bruker Avance 600 spectrometer equipped with a 5 mm ^1^HTXI probe (Bruker BioSpin, Rheinstetten, Germany) at 37°C for blood plasma and 25°C for urine. A standard Bruker “ZGPR” pulse program that applies a presaturation pulse sequence for water suppression was used, and a total of 64 scans were collected into 32 K data points with a relaxation delay of 2 sec. A spectral width of 7288.63 Hz and an acquisition time per scan of 2.25 sec were applied in this study. Prior to analysis, samples were thawed and homogenized using a vortex mixer. Urine samples were centrifuged at 10,000 g for 5 min to remove insoluble material, and 500 *μ*L supernatant was transferred to a 5 mm NMR tube and mixed with 100 *μ*L of a 0.75 M phosphate buffer solution (containing 0.5% sodium trimethylsilyl propionate-d_4_ (TSP)) prepared in D_2_O. Blood plasma samples were centrifuged at 10,000 g for 10 min and 400 *μ*L supernatant was transferred to a 5 mm NMR tube and then mixed with 200 *μ*L D_2_O.

### 2.3. Physical Activity

Physical activity was measured by means of a short questionnaire daily, wherein participants registered the number of counts measured by using pedometers (Yamax, SW-200) for 7 consecutive days [25, 26].

### 2.4. Tanner Stage

Tanner stage was determined on a 5-point scale according to an assessment of pubic hair development in boys and breast stage in girls by using self-reported questionnaires [25, 26].

### 2.5. Data Analysis

All ^1^H NMR spectra were automatically phased and baseline-corrected using Topspin 3.0 software (Bruker BioSpin, Rheinstetten, Germany). The ^1^H spectra of blood plasma were referenced to the anomeric signal of *α*-glucose at 5.23 ppm, while the ^1^H spectra of urine were referenced to the TSP signal at 0 ppm. Then, all spectra were aligned by using the “icoshift” procedure [28] in MATLAB (version R2012a, The Mathworks Inc., Natick, MA, USA). The spectral region from 0.0 to 10.0 ppm without the residual water resonance region from 4.7 to 5.0 ppm were normalized to the total signal intensities of the NMR spectra, subdivided into 0.01 ppm spectral regions, and integrated to 970 “bin” data for multivariate data analysis.

PCA and OPLS-DA for classification and PLSR for regression were performed on mean-centered and Pareto-scaled data by using the SIMCA 13.0 software (Umetrics, Umeå, Sweden), and a leave-one-out cross validation (LOOCV) method was used to determine the optimal number of latent variables for the models. *R* ^2^ *X* and *R* ^2^ *Y* are the percentage of the variance in *X* and *Y* matrixes explained by the current latent variable of the model, respectively, while *Q* ^2^ *Y* is the predictive capability of the model. In addition, the significance test of the model was performed by using CV-ANOVA [29] in the SIMCA software. Outliers in the models were identified as samples located far away from the 95% Hotelling's T_2_ confidence limit. For urine, 7 outliers were identified and excluded from the models resulting in a total of 185 samples from 115 girls and 70 boys. For blood, 3 outliers were identified and excluded from the models resulting in a total of 189 samples from 119 girls and 70 boys. The NMR peaks were assigned based on reported values [30, 31]. To aid spectral assignment, two-dimensional (2D) ^1^H-^1^H correlation spectroscopy with double-quantum filter (COSY), 2D ^1^H-^1^H total correlation (TOCSY) and 2D ^13^C-^1^H heteronuclear single quantum coherence (HSQC), experiments were performed on representative samples of both urine and plasma. For analysis of selected urinary metabolites, integration of the specified ppm area including citrate (CH_2_: 2.66–2.71 ppm), creatinine (CH_2_: 4.04–4.07 ppm), phenylacetylglutamine (CH: 7.33–7.39 ppm), hippurate (CH_2_-2,6: 7.81–7.86 ppm), and urea ((NH_2_)_2_: 5.50–6.10 ppm) in the NMR spectrum was performed by using Topspin 3.0 software, followed by calculation of the relative concentrations according to the known TSP concentration. A linear mixed effects model was performed on the relative concentration by using MIXED procedure in SAS 9.2 (SAS Institute Inc, Cary, NC) to evaluate effect of Tanner stage and gender on these metabolites. The mixed model included the fixed effects of gender, Tanner stage, and their interaction, while the intercept of model and individuals were used as a random effect. The restricted maximum likelihood (REML) approach [32] was used to estimate the variance components of models. The degrees of freedom were determined according to the method of Kenward and Roger [33], and Akaike Information Criterion [34] was performed to evaluate the optimal model. In addition, least square (LS) means procedures were used to calculate means and standard errors and pairwise *t*-tests for multiple comparisons were estimated by using the Tukey test. In this study, main and interaction effects were considered statistically significant when *P* < 0.05.

## 3. Results and Discussion

### 3.1. Inter-/Intrasubject Variations in the Urine and Blood Metabolomes over a 12-Week Period

**Figure 1:**
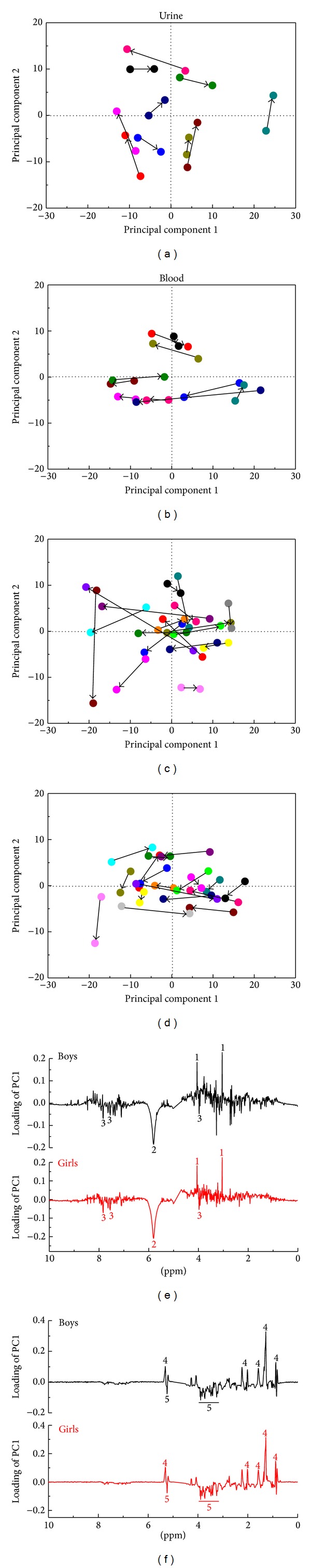
Intrasubject variation in the urine and plasma metabolome of adolescents over a 12-week interval: (a) PCA score plot of urine samples from boys (PC1 explains 28.3% and PC2 explains 16.4% of the variation); (b) PCA score plot of plasma samples from boys (PC1 explains 64.3% and PC2 explains 14.4% of the variation); (c) PCA score plot of urine samples from girls (PC1 explains 24.3% of the variation and PC2 explains 10.2% of the variation); (d) PCA score plot of plasma samples from girls (PC1 explains 55.7% and PC2 explains 11.6% of the variation); (e) PC1 loadings of urine samples; (f) PC1 loadings of plasma samples. The same subject is indicated by the same color in PCA score plots and the arrow points from week 0 to week 12. Assignments: 1: creatinine (3.04 and 4.05 ppm); 2: urea (5.50–6.10 ppm); 3: hippurate (3.96, 7.54, and 7.82 ppm); 4: lipids; 5: glucose.

PCA scores plots (Figure 1) show that, in many cases, the two samples obtained from the same subject are positioned close to each other; however, intrasubject variations of urine and plasma samples are still observed in both genders. A higher intersubject variation is observed for the urine metabolome than for the plasma metabolome (Figure 1), which is in agreement with previous studies [35, 36]. The corresponding loadings from urine (Figure 1(e)) and plasma (Figure 1(f)) samples were examined in order to elucidate the spectral regions most susceptible to inter-/intrasubject variations. The loadings revealed that signals at 3.04 and 4.05 ppm from creatinine and a broad signal from urea at 5.50–6.10 ppm contribute to intrasubject variation in urine metabolome, while minor contribution from hippurate signals at 3.96, 7.54, and 7.82 ppm is also evident (Figure 1(e)). Walsh et al. [36] have also concluded that hippurate and creatinine were the metabolites contributing most to variation in the urinary profiles. Figure 1(f) illustrates that lipids and glucose in plasma mainly contribute to inter-/intrasubject variation, which is in agreement with results from Lenz et al. [35]. Krug et al. [8] reported that the human metabolome is under continuous changes due to anabolic (after meal) and catabolic (during fasting or physical exercise) conditions of metabolism. Thus, the variations in the urine and plasma metabolome can be ascribed to a range of factors including dietary effects, physical exercise, and physiological stress.

### 3.2. Gender Differences in the Urine and Blood Metabolomes

**Figure 2:**
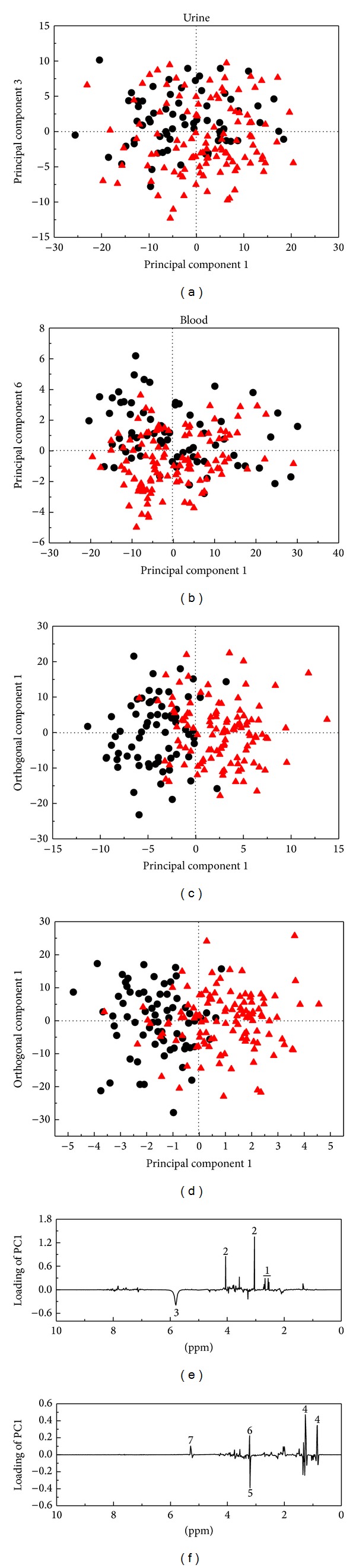
Gender difference (●: boys; ▲: girls) in the urine and plasma metabolome of adolescents: (a) PCA score plot of urine samples (PC1 explains 20.3% and PC3 explains 5.4% of the variation); (b) PCA score plot of plasma samples (PC1 explains 59.2% and PC6 explains 2.3% of the variation); (c) OPLS-DA score plot of urine samples (1 predictive and 2 orthogonal components; *R* ^2^ *X* = 32.8%; *R* ^2^ *Y* = 50.4%; *Q* ^2^ = 28.3%; *P* < 0.0001); (d) OPLS-DA score plot of plasma samples (1 predictive and 3 orthogonal components; *R* ^2^ *X* = 73.8%; *R* ^2^ *Y* = 46.4%; *Q* ^2^ = 26.5%; *P* < 0.0001); (e) S-line plot of urine samples; (f) S-line plot of plasma samples. Assignments: 1: citrate (2.53, 2.56, 2.67, and 2.70 ppm); 2: creatinine (3.04 and 4.05 ppm); 3: urea (5.50–6.10 ppm); 4: lipoproteins (0.84 and 1.26 ppm); 5: choline (3.20 ppm); 6: phosphocholine (3.22 ppm); 7: unsaturated lipids (5.29 ppm).

Gender was found to have a pronounced impact on the urine and plasma metabolomes, and O-PLS models that could discriminate the two gender could be built from the metabolomics data obtained both for urine (*R* ^2^ *X* = 32.8%; *R* ^2^ *Y* = 50.4%; *Q* ^2^ = 28.3%; *P* < 0.0001) (Figure 2(c)), which included samples from 115 girls and 70 boys, and plasma (*R* ^2^ *X* = 73.8%; *R* ^2^ *Y* = 46.4%; *Q* ^2^ = 26.5%; *P* < 0.0001) (Figure 2(d)), which included samples from 119 girls and 70 boys.

Inspection of the corresponding S-line plot from the urine data (Figure 2(e)) revealed that gender differences could be ascribed to differences in the urinary content of citrate (2.53, 2.56, 2.67, and 2.70 ppm), creatinine (3.04 and 4.05 ppm), and urea (5.50–6.10 ppm). Mixed model analysis shows that the concentrations of citrate and creatinine were significantly higher in girls, while urea content was lower in girls compared with boys (Table 1). The results are in agreement with previous studies on adults [3, 4, 37] where higher urinary levels of citrate have been observed in females compared with males. It has been reported that the excretion of urinary citrate is regulated by sex hormones such as estrogen [38] and testosterone [39], which may contribute to the gender differences in urinary citrate. However, the results concerning creatinine are opposing previous studies on adults who all reported that urinary creatinine levels were higher in males than females. A positive association between urinary excretion of creatinine and muscle mass has been reported by Kochhar et al. [3] and Oterdoom et al. [40]. Neu et al. [41] investigated the influence of puberty on muscle development and found that the gender difference in forearm muscle growth decreased until pubertal stage 3 and then increased again. Generally, girls begin and complete each puberty stage earlier than boys, which was also reflected in our study (Table 1). Thus, the contrasting results about urinary creatinine may be related to a higher muscle mass in girls relative to boys. Since urea is the major product of protein catabolism, the higher urinary urea excretion in boys may be caused by a higher protein intake or a higher protein turnover. A higher leucine oxidation in males than in females has been reported, which could cause the gender-specific difference in protein utilization [42, 43]. In addition, females may utilize less protein as an energy source owing to a greater part of exercise energy from fat [44]. In addition, Table 1 shows that girls have a relatively higher urinary excretion of hippurate and phenylacetylglutamine than boys, which is different from results obtained for adults aged 40–59 years [45]. They found a higher hippurate excretion in men compared to women, but no gender difference in phenylacetylglutamine excretion. Consequently, it appears that many of the gender effects observed on the urine metabolome are being commenced already in childhood, while some differences are still evident between adolescents and adults.

Inspection of the S-line plot for blood data shows that the discrimination of the two genders mainly can be ascribed to differences in lipoproteins (0.84 and 1.26 ppm), phosphatidylcholine (3.22 ppm), and unsaturated lipid (5.29 ppm) and lower levels of choline (3.20 ppm) in girls compared with boys (Figure 2(f)). Gender differences in blood lipids have also previously been reported for 17-year-old Scandinavians [5]. According to Brindle et al. [46], the region at 3.22 ppm can be assigned to –N(CH_3_)^3+^ groups in molecules, which contain the choline moiety, mostly phosphatidylcholine from HDL. The –N(CH_3_)^3+^ groups could also be coupled with a higher level of unsaturated lipids in the blood from girls, implying that the phospholipids are more unsaturated in girls compared to boys [5]. In addition, phosphatidylcholine is a major structural constituent of cell membranes. Thus, our results could possibly reflect that girls have a higher overall plasma membrane turnover compared with boys [5]. A study on mice also found gender differences in phosphatidylcholine [47]. A survey in 2007-2008 estimated mean daily intake of choline in US population and reported that males have a higher choline requirement than females for all age groups above 12 years old [48], which could possibly explain the higher content of choline in the plasma from boys.

### 3.3. Effect of Pubertal Development Stage on the Urine and Blood Metabolomes

**Figure 3:**
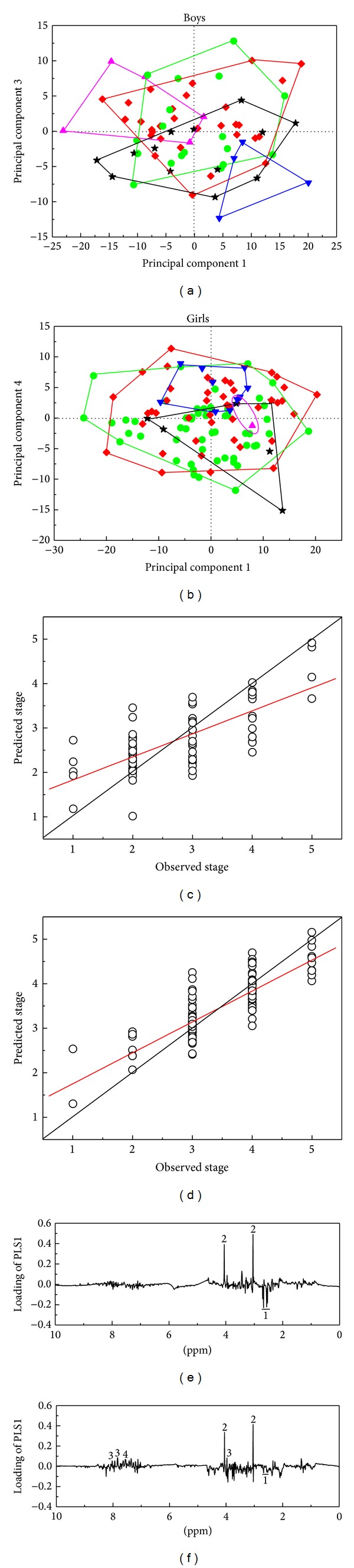
(a) PCA score plot of boys (PC1 explains 21.4% and PC3 explains 6.3% of the variation); (b) PCA score plot of girls (PC1 explains 19.7% and PC4 explains 5.9% of the variation) obtained for the urine metabolome of adolescents and showing Tanner stage (▲: stage 1; ♦: stage 2; ●: stage 3; ★: stage 4; *▼*: stage 5). (c) PLSR prediction plot of Tanner stage in boys (the optimal number of PLSs = 2; *R* ^2^ *X* = 27.1%; *R* ^2^ *Y* = 51.9%; *Q* ^2^ = 25.5%; *P* < 0.001); (d) PLSR prediction plot of Tanner stage in girls (the optimal number of PLSs = 4; *R* ^2^ *X* = 37.3%; *R* ^2^ *Y* = 69.4%; *Q* ^2^ = 24.6%; *P* < 0.0001); (e) PLS1 loadings of boys; (f) PLS1 loadings of girls. Assignments: 1: citrate (2.53, 2.56, 2.67, and 2.70 ppm); 2: creatinine (3.04 and 4.05 ppm); 3: hippurate (3.96, 7.54 and 7.82 ppm); 4: phenylacetylglutamine (7.35 ppm).

PCA of the NMR urine profiles of 115 girls and 70 boys indicated a tendency for a clustering according to Tanner stage (Figures 3(a) and 3(b)), revealing that Tanner stage is reflected in the urine metabolome. PLS models with Tanner stage as response variable were therefore constructed in order to elucidate the relation between urine metabolite profile and Tanner stage for boys (*R* ^2^ *X* = 27.1%; *R* ^2^ *Y* = 51.9%; *Q* ^2^ = 25.5%; *P* < 0.001) and girls (*R* ^2^ *X* = 37.3%; *R* ^2^ *Y* = 69.4%; *Q* ^2^ = 24.6%; *P* < 0.0001), respectively (Figures 3(c) and 3(d)). The corresponding PLS1 loadings indicate that urinary creatinine excretion is positively correlated with Tanner stage in both boys (Figure 3(e)) and girls (Figure 3(f)), which is supported by results from quantification of creatinine (Table 1). Oterdoom et al. [40] and Wang et al. [49] found a positive association between muscle mass and urinary excretion of creatinine, and increases in muscle mass increases during pubertal development is probably encompassed in the present findings. In addition, a study on creatinine levels surveyed a large US population with ages ranging from 6 to 70 years reported a gradual increase in urinary creatinine concentration up to an age between 20 and 29 years [50].

Intriguingly, a negative correlation is observed between urinary citrate excretion and Tanner stage, which is more evident in boys than in girls (Figures 3(e) and 3(f); Table 1). It has been reported that the flux of citrate through the TCA cycle is regulated by gender hormones such as testosterone [39]. Costello et al. [51] also found that citrate oxidation in rat ventral prostate was stimulated by testosterone. In addition, a strong correlation between estrogen actions and citrate excretion was reported by Dey et al. [38], who found that estrogen replacement increased urinary citrate excretion in postmenopausal women. Therefore, the decrease in the excretion of citrate is most likely attributed to changes in sex hormones during pubertal development.

Loading plots indicate that the aromatic region of ^1^H NMR spectra (6.80–8.10 ppm) involving mainly hippurate and phenylacetylglutamine to some extent was correlated with Tanner stage, especially for girls (Figure 3(f)). Quantification of hippurate and phenylacetylglutamine by integration of the NMR signals revealed no significant effect of Tanner stage on urinary hippurate, while phenylacetylglutamine tended to be significantly affected by Tanner stage (*P* = 0.06) (Table 1). Gu et al. [9] reported that urinary hippurate excretion may vary less during childhood development, although a relatively large variation in hippurate with age was found for adults by Psihogios et al. [37]. In addition, the data reported by Swann et al. [52] indicated that urinary phenylacetylglutamine concentrations were increased with age in adults. Urinary hippurate and phenylacetylglutamine have often been related to the activity of the gut microflora [53], so our results suggest that the gut microflora may vary with Tanner stage. Thus, further studies including a more detailed description of the gut microflora as function of pubertal development could be of great interest confirming this hypothesis. For blood, a relatively low correlation was obtained between the metabolite profile and Tanner stage (*R* ^2^ *Y* = 40.6% and *Q* ^2^ = 23.9% for boys; *R* ^2^ *Y* = 33.6% and *Q* ^2^ = 16.0% for girls) (Figure S1,See the Supplementary Material available online at http://dx.doi.org/10.1155/2014/537157).

### 3.4. Effect of Physical Activity on the Urine and Blood Metabolomes

Several studies have reported the use of NMR-based metabolomics to study impact of physical exercise on the biofluid metabolome [54–59]. In the present study the relation between the metabolome and physical activity measured as number of steps taken daily was elucidated. No strong correlation could be identified neither between the blood plasma nor the urine metabolome and daily physical activity (Figure  S2, Supplementary Material). Thus, it was not possible to demonstrate a relation between moderate physical activity and the metabolome. Possibly more extreme variations in physical activity would have a more clear effect on the metabolome; however, this remains to be established in future studies.

## 4. Conclusions

In summary, our findings showed that creatinine, hippurate, and urea in urine and glucose and lipids in plasma are the main metabolites giving rise to inter-/intrasubject variations. We showed that girls have a higher urinary excretion of citrate, creatinine, hippurate, and phenylacetylglutamine and higher plasma level of phosphatidylcholine and unsaturated lipid as compared with boys. In addition, we have identified potential metabolites including creatinine and citrate, which exhibit a relation to pubertal development stage as measured by the Tanner stage. To our knowledge, the present study is the first to elucidate the relation between the metabolome and pubertal development stage.

## Supplementary Material

Two additional figures supporting our results are provided. Figure S1 shows the results of PCA for classification among different Tanner stages and PLSR for correlation between Tanner stages and the metabolome in the plasma data from boys and girls. Figure S2 illustrates the relationship between observed and predicted physical activities by PLSR based on the urine and plasma metabolomes of adolescents and the corresponding loading plots of PLSR.

## Figures and Tables

**Table 1:** Mixed models of Tanner stage and gender effects in overweight adolescents on urinary metabolites^a^.

		*N* ^b^	Citrate	Creatinine	Hippurate	Phenylacetylglutamine	Urea
Gender	♂	70	27.1 ± 2.4^b^	262.9 ± 5.1^b^	46.6 ± 3.5^b^	38.9 ± 2.4	1358.2 ± 48.4^a^
	♀	115	36.4 ± 2.7^a^	280.0 ± 5.4^a^	57.1 ± 3.9^a^	45.4 ± 2.6	1087.3 ± 53.5^b^

Tanner stage	1	♂: 5; ♀: 2	49.2 ± 6.8^a^	262.8 ± 12.6^b^	39.6 ± 9.0	33.7 ± 6.1	1326.5 ± 124.0
	2	♂: 30; ♀: 7	29.7 ± 2.5^bc^	262.3 ± 6.9^b^	57.0 ± 5.0	41.2 ± 3.3	1211.8 ± 67.9
	3	♂: 18; ♀: 53	30.8 ± 1.7^b^	257.7 ± 4.3^b^	57.4 ± 3.0	47.5 ± 2.2	1256.6 ± 43.1
	4	♂: 13; ♀: 43	25.1 ± 1.9^c^	270.4 ± 6.6^b^	56.6 ± 3.8	48.3 ± 2.6	1257.8 ± 54.7
	5	♂: 4; ♀: 10	24.1 ± 4.6^bc^	303.8 ± 8.9^a^	48.5 ± 6.4	40.0 ± 4.3	1060.9 ± 87.7

Significant effects (*P* values)	G^c^		**0.01**	**0.02**	**0.05**	0.07	**0.0002**
	T^d^		**0.005**	**0.0003**	0.30	0.06	0.29
	G ∗ T		0.24	0.81	0.73	**0.009**	**0.03**

^a^Values are the relative concentrations according to a known TSP concentration and expressed mean ± SE; ^b^Number of subjects (♂: boys; ♀: girls); ^c^Gender; ^d^Tanner stages; Different letters indicate significant differences within columns (*P* < 0.05).

## List of Abbreviations

BMI: Body mass index
COSY: Two-dimensional ^1^H-^1^H correlation spectroscopy with double-quantum filter
CVD: Cardiovascular disease
CV-ANOVA: Cross validation-analysis of variance
HDL: High-density lipoprotein
HSQC: Two-dimensional ^13^C-^1^H heteronuclear single quantum coherence
LS: Least squares
LOOCV: Leave-one-out cross validation
NMR: Nuclear magnetic resonance
O-PLS: orthogonal-partial least squares
OPLS-DA: Orthogonal partial least squares-discriminant analysis
PCA: Principal component analysis
PLS: Partial least squares
PLSR: Partial least squares regression
*Q*^2^*Y*: The predictive capability of the model
*R*^2^*X*: The percentage of the variance in *X* matrixes explained by the current latent variable of the model
*R*^2^*Y*: The percentage of the variance in *Y* matrixes explained by the current latent variable of the model
REML: Restricted maximum likelihood
TMAO: Trimethylamine-N-oxide
TSP: Sodium trimethylsilyl propionate-d_4_
TCA: Tricarboxylic acid
TOCSY: Two-dimensional ^1^H-^1^H total correlation
wk: Week
ZGPR: Standard Bruker water presaturation sequence.
