# Supplementary material for: NMR-Based Metabolomic Profiling of Overweight Adolescents: An Elucidation of the Effects of Inter-/Intraindividual Differences, Gender, and Pubertal Development
Source: Biomed Res Int. 2014 Mar 27;2014:537157. doi: 10.1155/2014/537157 (PMC3985195; doi:10.1155/2014/537157)
Supplement: Supplementary file 1 — Two additional figures supporting our results are provided. Figure S1 shows the results of PCA for classification among different Tanner stages and PLSR for correlation between Tanner stages and the metabolome in the plasma data from boys and girls. Figure S2 illustrates the relationship between observed and predicted physical activities by PLSR based on the urine and plasma metabolomes of adolescents and the corresponding loading plots of PLSR. [file 537157.f1.docx]

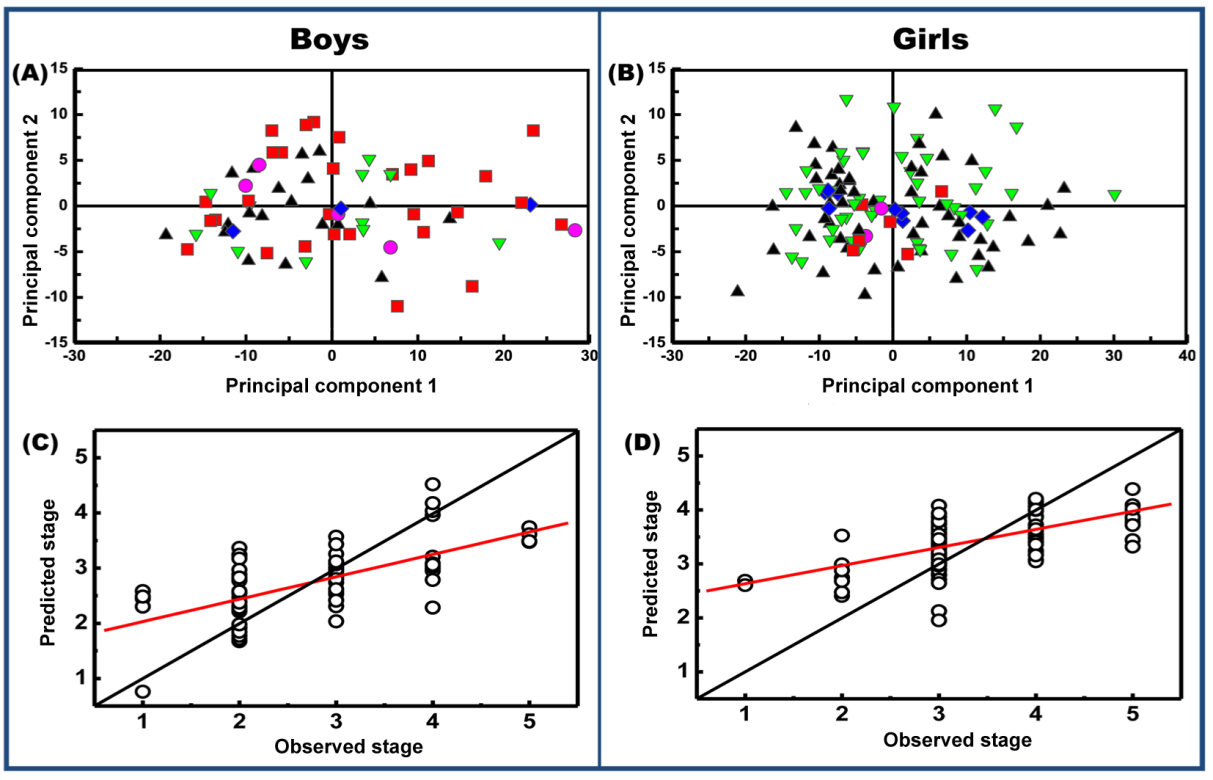

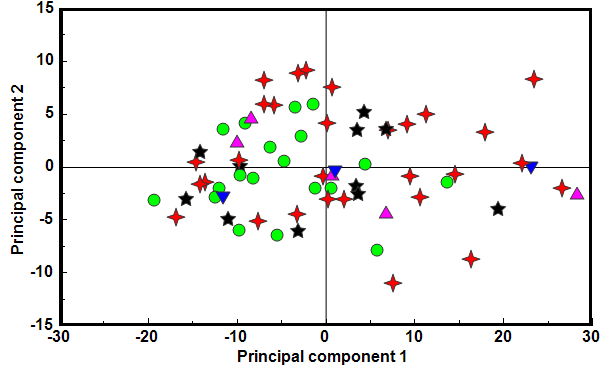

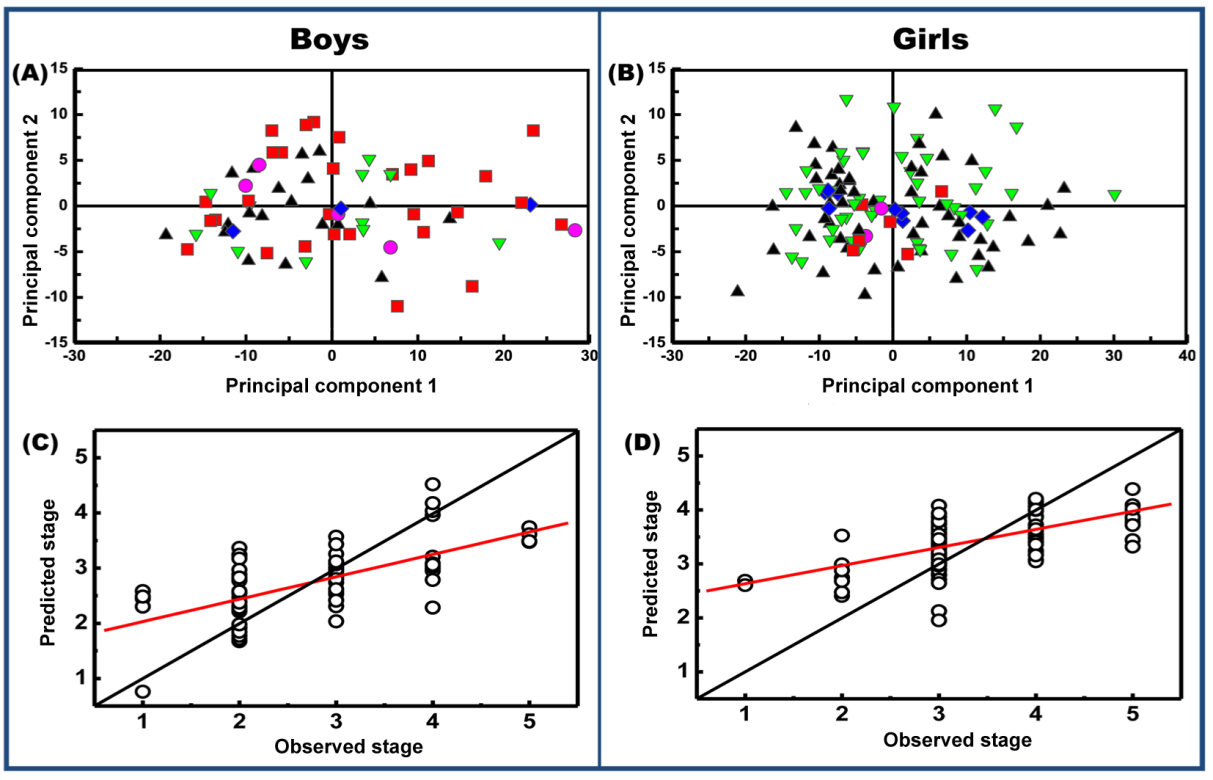

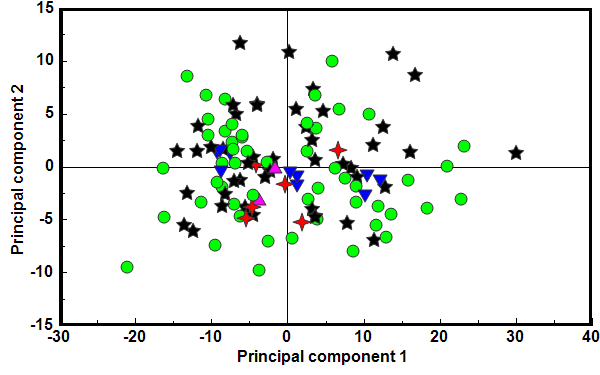


**(A)**

**(B)**

**Boys**

**Girls**

**FIGURE. S1** Tanner stages (▲, stage 1; 🟆, stage 2; ●, stage 3; 🟊, stage 4; ▼, stage 5) in the plasma metabolome of adolescents: (A) PCA score plot of boys (R^2^X_[1]_=65.1%; R^2^X_[2]_=9.9%); (B) PCA score plot of girls (R^2^X_[1]_=56.7%; R^2^X_[2]_=11.9%); (C) PLSR prediction of Tanner stages in boys (The optimal number of PLSs=3; R^2^X=81.4%; R^2^Y=40.6%; Q^2^=23.9%; P<0.05); (D) PLSR prediction of Tanner stages in girls (The optimal number of PLSs=3; R^2^X=72.2%; R^2^Y=33.6%; Q^2^=16.0%; P<0.05).

**
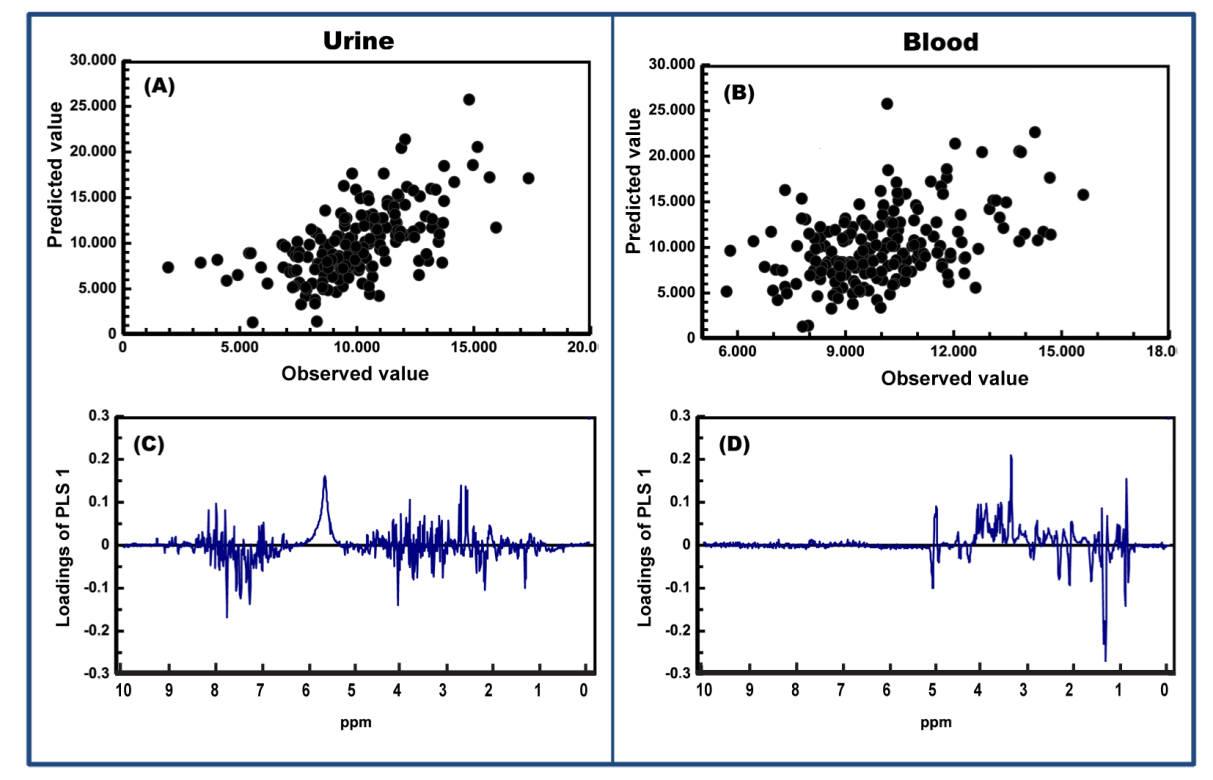
**

**FIGURE. S2** Correlation between physical activities and the urine and plasma metabolomes of adolescents: (A) PLSR based on urine samples (The optimal number of PLSs=3; R^2^X=30.8%; R^2^Y=36.3%; Q^2^=5.72%; P=0.03); (B) PLSR based on plasma samples (The optimal number of PLSs=3; R^2^X=72.9%; R^2^Y=20.6%; Q^2^=6.02%; P=0.04); (C) PLS 1 loadings of urine samples; (D) PLS 1 loadings of plasma samples.
